# Induction of Jasmonoyl-Isoleucine (JA-Ile)-Dependent *JASMONATE ZIM-DOMAIN* *(JAZ)* Genes in NaCl-Treated *Arabidopsis thaliana* Roots Can Occur at Very Low JA-Ile Levels and in the Absence of the JA/JA-Ile Transporter JAT1/AtABCG16

**DOI:** 10.3390/plants9121635

**Published:** 2020-11-24

**Authors:** Corinna Thurow, Markus Krischke, Martin J. Mueller, Christiane Gatz

**Affiliations:** 1 Department of Plant Molecular Biology and Physiology, Albrecht-von-Haller Institute for Plant Sciences, University of Göttingen, Julia-Lermontowa-Weg 3, 37077 Göttingen, Germany; cthurow@gwdg.de; 2 Pharmaceutical Biology, Julius-von-Sachs-Institute for Biosciences, University of Würzburg, 97082 Würzburg, Germany; markus.krischke@biozentrum.uni-wuerzburg.de (M.K.); martin.mueller@biozentrum.uni-wuerzburg.de (M.J.M.)

**Keywords:** allene oxide synthase, CORONATINE INSENSITIVE 1, jasmonoyl-isoleucine, JA/JA-Ile transport protein JAT1, roots, salt

## Abstract

The plant hormone jasmonoyl-isoleucine (JA-Ile) is an important regulator of plant growth and defense in response to various biotic and abiotic stress cues. Under our experimental conditions, JA-Ile levels increased approximately seven-fold in NaCl-treated *Arabidopsis thaliana* roots. Although these levels were around 1000-fold lower than in wounded leaves, genes of the JA-Ile signaling pathway were induced by a factor of 100 or more. Induction was severely compromised in plants lacking the JA-Ile receptor CORONATINE INSENSITIVE 1 or enzymes required for JA-Ile biosynthesis. To explain efficient gene expression at very low JA-Ile levels, we hypothesized that salt-induced expression of the JA/JA-Ile transporter *JAT1/AtABCG16* would lead to increased nuclear levels of JA-Ile. However, mutant plants with different *jat1* alleles were similar to wild-type ones with respect to salt-induced gene expression. The mechanism that allows COI1-dependent gene expression at very low JA-Ile levels remains to be elucidated.

## 1. Introduction

Plant hormones serve as signaling molecules that coordinate growth and stress responses depending on internal or external cues. It is generally accepted that hormone signaling starts with the increased abundance of the active hormone, so the incidences of hormone-associated receptors become more frequent. Upon hormone binding, receptors alter their activities, causing, in many cases, transcriptional reprogramming. Well-established jasmonate-induced signaling processes follow this concept, with wounding or other forms of tissue damage leading to elevated levels of (+)-7-*iso*-jasmonic acid (JA) and its derivatives, including the active hormone jasmonoyl-isoleucine (JA-Ile) (for recent reviews, see [1,2]). At low JA-Ile levels, the JASMONATE ZIM-DOMAIN (JAZ) family of repressor proteins interferes with the activity of various transcription factors, including MYC2 [3,4]. Upon JA-Ile accumulation in the cytosol, the ATP binding cassette (ABC) transporter JAT1/AtABCG16 facilitates JA-Ile transport into the nucleus [5]. Here, JA-Ile binds to the F-box protein CORONATINE INSENSITIVE 1 (COI1), which is part of the E3 ubiquitin ligase SCF^COI1^ [6]. The F-box domain of COI1 interacts with the mediator subunit Mediator Complex Subunit 25 (MED25), facilitating its recruitment to promoters that are repressed by JAZ proteins [7]. JA-Ile promotes the formation of a transient ternary complex between COI1 and JAZ proteins, resulting in ubiquitination of JAZs and their subsequent degradation through the 26S proteasome. Decreasing levels of JAZ proteins lead to the activation of target genes [8,9]. 

In *Arabidopsis thaliana*, at least a subfraction of COI1-dependent genes are not only induced upon tissue damage but also upon various forms of abiotic stress, including salt treatment [10,11,12], osmotic stress [13], drought [14], application of xenobiotics, extended darkness [15] or fumigation with NO_2_ [16]. Increased JA and/or JA-Ile levels have been observed in plants watered with NaCl [17] or subjected to drought [17,18] or osmotic stress [13,19], while no increase in JA or JA-Ile was detected in leaves treated with xenobiotic chemicals or exposed to extended darkness or NO_2_ [15,20]. How COI1 is activated in the latter three cases has remained obscure.

Incidental observations of very low JA-Ile levels in salt-treated roots, in combination with information from public databases that transcription of the JA/JA-Ile transporter *JAT1* is highly induced upon salt treatment, prompted us to ask the question of whether JAT1 might compensate for low JA-Ile levels. Here, we show that induction of selected COI1- and JA-Ile-dependent genes in salt-treated roots can occur in the presence of JA-Ile levels that are 1000-fold lower than in wounded leaves. Unexpectedly, JAT1 was not required for activation of gene expression. The mechanism of the high sensitivity of JA-Ile-dependent signaling in salt-treated roots and other tissues remains to be elucidated.

## 2. Results

**Figure 1:**
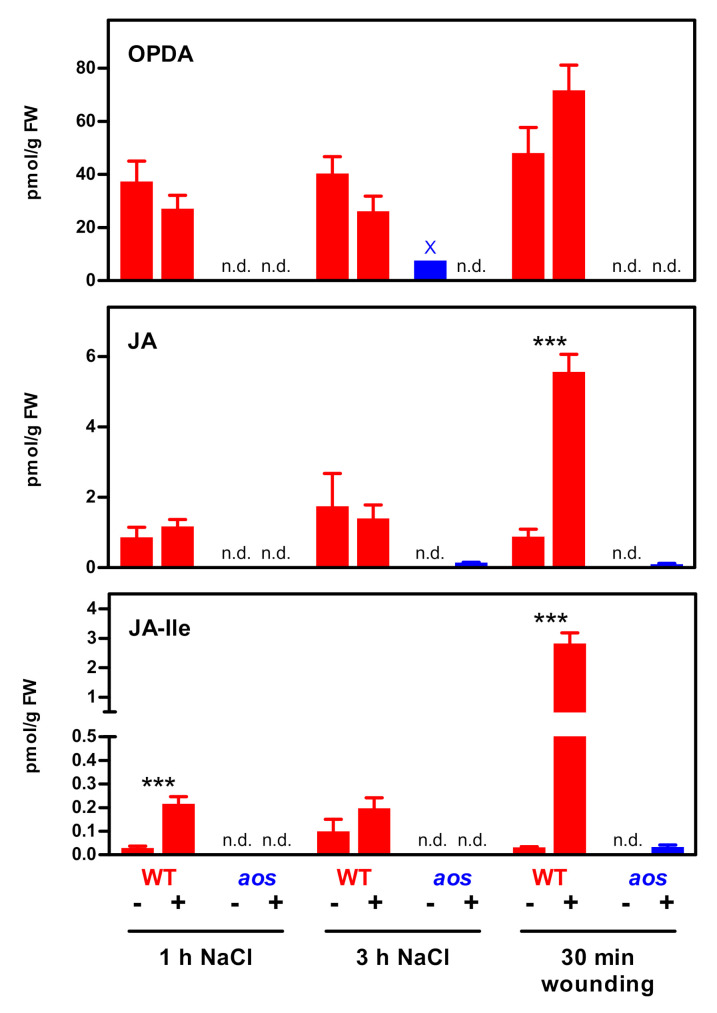
The *cis*-(+)-12-oxo phytodienoic acid (OPDA), (+)-7-*iso*-jasmonic acid (JA) and jasmonoyl-isoleucine (JA-Ile) levels in roots of *Arabidopsis thaliana* wild-type and *allene oxide synthase* (*aos)* plants upon NaCl treatment and after wounding. Data are mean values ± SEM of four (wild-type) or three (*aos*) samples, each representing pooled root tissue from two individual plants. Asterisks denote statistical significance between control and treated samples (unpaired, two-tailed *t*-test. *** *p* < 0.001). The OPDA value for the three-hour NaCl treatment of *aos* plants (marked with a cross) was obtained from one sample, whereas no OPDA was detected in the other two extracts. n.d.; not detected.

Jasmonate levels in roots in response to salt stress were determined in *Arabidopsis* plants grown in a hydroponic culture (Supplementary Figure S1). Six-week-old plants were treated for one or three hours with 150 mM sodium chloride (NaCl). The jasmonates *cis*-(+)-12-oxo phytodienoic acid (OPDA), JA and JA-Ile were analyzed in roots by ultra-performance liquid chromatography-electrospray tandem mass spectrometry (UPLC-ESI–MS/MS) (Figure 1). While OPDA and JA levels did not increase, seven-fold elevated JA-Ile amounts were detected after one hour of NaCl treatment. JA and JA-Ile levels were 2.8- (JA) or 13-fold (JA-Ile) lower than in wounded roots. At three hours, JA-Ile levels in mock-treated plants were statistically not different from the levels observed in NaCl-treated plants, which is mainly due to one enhanced value which had 0.2 pmol/mg fresh weight (FW), while the values of the other three samples were either below the detection level or contained 0.04 or 0.06 pmol/g FW. Values were close to background but clearly different from those measured in the jasmonate biosynthesis mutant *allene oxide synthase* (*aos*). Very low JA or JA-Ile values were occasionally found in the *aos* mutant at 3 h of NaCl treatment or after wounding, suggesting a minor *aos* activity due to unknown either enzymatic or non-enzymatic activities. Increased levels of the weak COI1 ligand 12OH-JA-Ile [21,22] were observed in roots upon wounding and in leaves of salt-treated plants, but not in salt-treated roots (Supplementary Figure S2).

**Figure 2:**
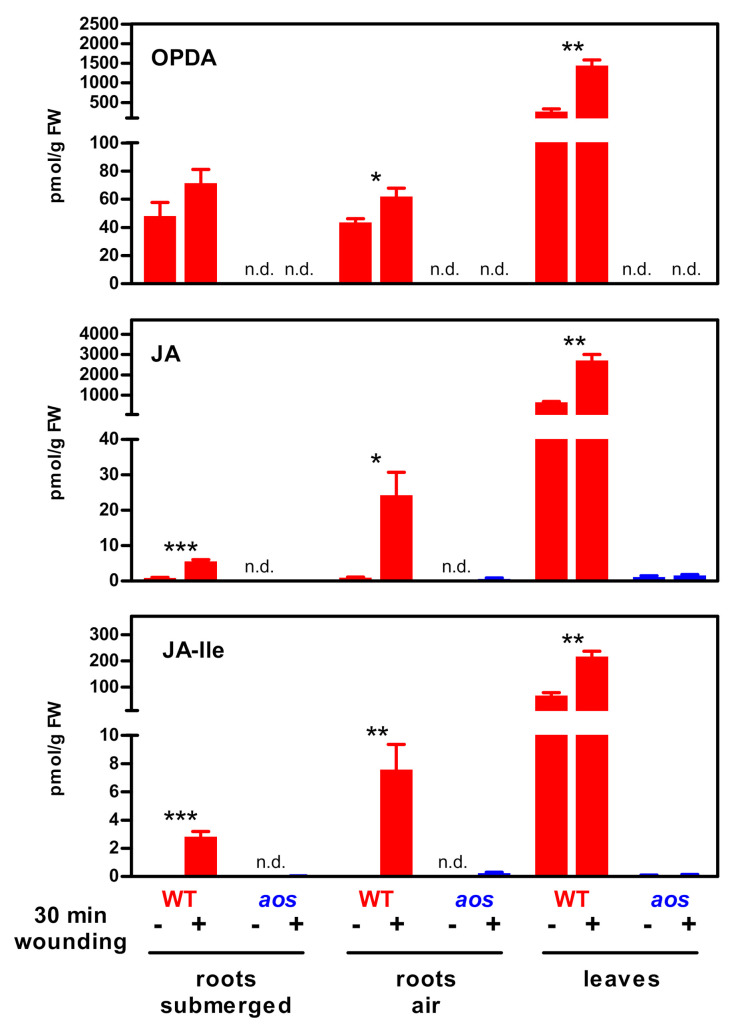
OPDA, JA and JA-Ile levels in roots and leaves of wild-type and *aos* plants, 30 min after wounding (+) or control treatment (−). Roots were either incubated in hydroponic solution (submerged, same samples as shown in Figure 1) or air (pseudo-aeroponic). Data are mean values ± SEM from four (wild-type roots) or three (wild-type leaves and *aos* roots and leaves) samples, each obtained from two pooled individual plants. Asterisks denote statistical significance between control and treated samples (unpaired, two-tailed *t*-test. * *p* < 0.05; ** *p* < 0.01; *** *p* < 0.001). n.d.; not detected.

The low levels of all oxylipins prompted us to ask whether these metabolites might leak into the 15-L hydroponic container. To address this question, roots were wounded and cultivated for 30 min, either in the hydroponic system or in an emptied container with some liquid (1 cm) left at the bottom (pseudo-aeroponic conditions). In this system, induced JA and JA-Ile amounts were found to be, by a factor of 4.4 (JA) or 2.7 (JA-Ile), higher than in the hydroponic system (Figure 2). Still, wounded roots cultivated under pseudo-aeroponic conditions contained approximately 30-fold lower amounts of JA-Ile than wounded leaves, indicating that differences in JA-Ile levels in roots as compared to shoots are not due to massive leakage.

Despite the low amounts of JA-Ile, transcript levels of primary JA-responsive genes (*JAZ5*, *JAZ7* and *JAZ10*) were induced in the root material collected for the analysis shown in Figure 1 (Supplementary Figure S3). Although JA-Ile levels were more than ten-fold lower in the NaCl-exposed wild-type roots than in the wounded roots, the expression of *JAZ5* and *JAZ10* was in the same range. Only *JAZ7* transcript levels were higher upon wounding.

**Figure 3:**
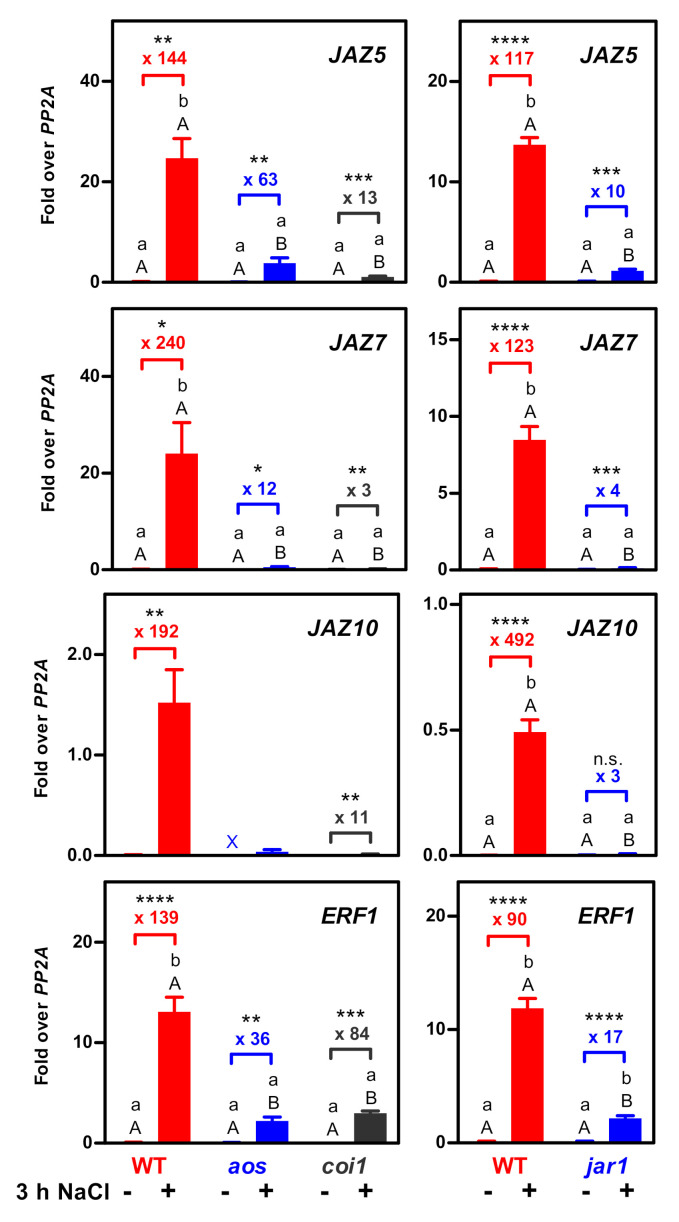
Quantitative RT-PCR analysis of *JAZ5*, *JAZ7*, *JAZ10* and *ERF1* transcript levels in root material of the indicated genotypes after three hours of either control or NaCl treatment. Relative transcript levels were determined using *PP2A* as a reference gene. The mean values (± SEM) obtained from three to five individually harvested plants are shown. The *JAZ10* value for the control treatment of *aos* plants (marked with a cross) was obtained from one sample only; the other values were below the detection limit. Numbers indicate fold change values after salt treatment and asterisks denote statistical significance when each genotype was examined separately (unpaired, two-tailed *t*-test. * *p* < 0.05; ** *p* < 0.01; *** *p* < 0.001; **** *p* < 0.0001; n.s., not significant). Overall statistical analysis was done using a two-way ANOVA followed by Bonferroni’s post-test: uppercase letters indicate significant differences (*p* < 0.05) between genotypes subjected to the same treatment; lowercase letters indicate significant differences (*p* < 0.05) between control and NaCl treatments performed with the same genotype.

At least in leaves, many JA-responsive genes can also be induced by abscisic acid (ABA) [14]. Because of the very low JA-Ile levels in roots, and since ABA increased in salt-treated roots (Supplementary Figure S2), we wondered whether JA, JA-Ile and COI1 were required for increased gene expression. To this end, transcript levels of *JAZ5*, *JAZ7* and *JAZ10* were quantified in root material collected from NaCl- and mock-treated wild-type *aos*, *coi1* and *jar1* plants (Figure 3). No significant differences (*p* < 0.05) between control and NaCl treatments were observed in *coi1*, *aos* and *jar1* in statistical analysis using all genotypes and treatments (Figure 3). This indicates that COI1 and JA-Ile are essential for the induction of *JAZ5*, *JAZ7* and *JAZ10* in salt-treated roots. A similar phenomenon—e.g., induction of *JAZ* expression at very low JA-Ile levels in WT but not in *aos* and *coi1*—had been observed before in leaves treated with xenobiotic 2,3,5-triiodobenzoic acid (TIBA) (Supplementary Figure S4 and [15]).

In a previous study, *JAZ* genes had been shown to be significantly induced in salt-treated *coi1* and *-2* roots, with background and induced transcript levels both being lower than in wild-type plants [23]. These results might suggest that COI1 would constitutively enhance basal and induced gene expression. Under our experimental conditions, basal *JAZ* expression was also lower in untreated *aos* and *coi1* plants (Supplementary Figure S5). Since *JAZ* gene expression is known to be promoted by COI1- and JA-Ile-dependent degradation of JAZ proteins, we conclude that even very low basal JA-Ile levels are sufficient to mediate a certain turn-over of JAZ proteins. When calculating induction factors, we found that the fold change was clearly lower in *coi1* than in the wild-type (Figure 3). Apparently, the E3 ligase activity of SCF^COI1^ acting on JAZ proteins increases in the wild-type after salt treatment. The fold change values were also reduced in *aos* and *jar1*, which is consistent with the notion that mutations in the receptor and its ligand should have the same phenotype. Unexpectedly, the two JA-Ile biosynthesis mutants *aos* and *jar1* show a difference with respect to *JAZ5* expression. This might indicate that metabolites generated in *aos* but not in *jar1* can induce *JAZ5* in a COI1-dependent manner. 

A slightly different picture is seen with *ETHYLENE RESPONSE FACTOR1* (*ERF1*), which serves as an upstream regulator of a variety of genes counteracting salt or drought stress [24]. As compared to the expression of the selected *JAZ* genes, *ERF1* expression and the fold induction are not as severely affected by *coi1*, *aos* and *jar1*. This is most likely due to the fact that *ERF1* expression is also influenced by the ethylene pathway [25,26]. Here, the stability of the upstream master regulator ETHYLENE INSENSITIVE3 (EIN3) is regulated by the ethylene signaling cascade [27]. In addition, EIN3 interacts with JAZ repressor proteins, which explains the integration of JA and ethylene signaling [28].

**Figure 4:**
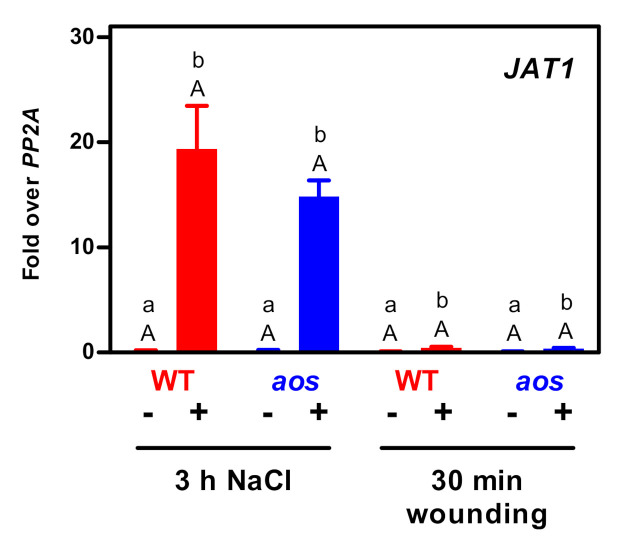
Quantitative RT-PCR analysis of *JAT1* transcript levels in RNA collected from root material of wild-type and *aos* plants under control conditions (−), upon NaCl treatment for three hours (+) or after wounding with subsequent incubation for 30 minutes in hydroponic solution (+). Relative transcript levels were determined using *PP2A* as a reference gene. Data are mean values ± SEM of three to four samples, each representing pooled root tissue from two individual plants. Statistical analysis was performed for the different inductions separately using a two-way ANOVA followed by Bonferroni’s post-test: uppercase letters indicate significant differences (*p* < 0.05) between genotypes subjected to the same treatment; lowercase letters indicate significant differences (*p* < 0.05) between control and treatment performed with the same genotype.

The activation of the JA-Ile signaling cascade at very low JA-Ile levels raised the question of whether other mechanisms arrange for higher local levels of the ligand at the site of receptor action. Previously, the JA-inducible ABC transporter JAT1/AtABCG16 was shown to be important for a variety of JA-induced responses. JAT1/ABCG16 facilitates the transport of JA across the plasma membrane and the transport of JA-Ile into the nucleus [5], where COI1 and JAZ are located. Interestingly, transcript levels of *JAT1/AtABCG16* increased in roots treated with salt for three hours (Figure 4). Induction was independent from *aos* and, thus, from JA. Only slight induction in *aos* and WT plants was observed upon wounding.

**Figure 5:**
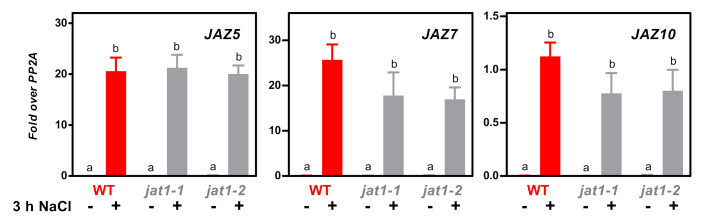
Quantitative RT-PCR analysis of *JAZ5*, *JAZ7* and *JAZ10* transcripts in root material from wild-type plants and two different *jat1* T-DNA insertion mutants after three hours of either control or NaCl treatment. Relative transcript levels were determined using *PP2A* as a reference gene. The mean values (± SEM) obtained from roots from four to five individually harvested plants are shown. Statistical analysis was done using a two-way ANOVA followed by Bonferroni’s post-test: lowercase letters indicate significant differences (*p* < 0.05) between control and NaCl treatment performed with the same genotype; there are no significant differences between genotypes subjected to the same treatment. Reduced *JAT1* transcript levels in the *jat1* mutants are depicted in Supplementary Figure S6.

We therefore hypothesized that increased abundance of JAT1/AtABCG16 in the nuclear envelope might lead to a local enrichment of JA-Ile in the nucleus. However, this hypothesis was not supported by experiments performed with two *jat1* mutants. Contrary to our expectations, NaCl-induced expression of the *JAZ* genes in *jat1-1* and *jat1-2* was not different from wild-type plants (Figure 5).

## 3. Discussion

Here, we report that JA signaling genes were induced in roots upon NaCl treatment, although induced JA-Ile levels were even 1000-fold lower than in wounded leaves. We rejected the hypothesis that salt-induced expression of JAT1/ABCG16, an ABC transporter that facilitates JA transport across the plasma membrane and nuclear accumulation of JA-Ile (5), would compensate for low JA-Ile biosynthesis by increasing the local JA-Ile concentrations at the site of action of the JA-Ile receptor, COI1.

### 3.1. The JA Pathway Can Be Activated Without a Strong JA-Ile Increase

In general, timing and amplitude of the jasmonate responses correlate with JA-Ile accumulation, which fits the concept that increased amounts of ligand receptor complexes lead to activation of the signaling cascade. However, more and more exceptions are being reported for the JA pathway. In leaves treated with the xenobiotic TIBA, for instance, *JAZ10* expression is induced in a manner that requires COI1 and JA biosynthesis (Supplementary Figure S4), although no increase in JA-Ile levels was detected [15]. Likewise, expression of several *JAZ* genes was induced by a factor of at least 30 in plants that had been harvested immediately after exposure to gaseous NO_2_ [16]. At this time point, no significant increase in JA-Ile or the weak COI1-ligand 12OH-JA-Ile was detected. Whether expression is COI1- or JA-Ile-dependent has not been tested, but it seems very likely that it is. *JAZ* genes belong to the primary response genes as shown by chromatin immunoprecipitation (ChIP) with sequencing (ChIP-seq) analysis which had revealed that all thirteen *JAZ* promoters are bound by MYC2 and/or MYC3 [29]; MYC2 and its related factors MYC3 and MYC4 are repressed when *JAZ* proteins are exceeding a certain threshold [30]. COI1-mediated degradation leads to MYC2-activated JAZ expression to prevent over-excitation of the pathway. Therefore, *JAZ* gene expression can be regarded as a good proxy for canonical JA signaling. 

### 3.2. Potential Artefacts of Low JA-Ile Measurements in Salt-Treated Roots

Still, we have to mention that previous reports have not observed a large difference in wound-induced JA-Ile levels in shoots vs. roots (13). We cannot entirely rule out that NaCl treatment facilitates leakage of oxylipins into the 15-L tank or that the extraction method is far less suited for roots as opposed to leaves. Moreover, it has to be considered that potentially higher local JA-Ile levels accumulate in specific cell types, although previous studies did not point at JA-Ile synthesis and signaling in only a few cells of the root. First, salt-induced degradation of a JAZ1:ß-GLUCURONIDASE fusion protein was observed in the meristematic zone and in the vascular tissue [23]; second, analysis of salt-induced gene expression after cell sorting implicates activation of the pathway mainly in all cell layers, except for the epidermis [10]. Since those studies were conducted with seedlings grown on a solid surface, while our study was performed with 6-week-old plants grown in hydroponic solution, we cannot entirely rule out the hypothesis of local high concentrations of the ligand. 

### 3.3. The JA/JA-Ile Transporter JAT1 Is Not Required for Salt-Induced Expression of JAZ Genes

Assuming that the increase in JA-Ile from 0.03 to 0.2 pmol/g FW is the crucial step for the activation of COI1 and that the pathway is not restricted to specific cells, we explored the hypothesis that the JA/JA-Ile transporter JAT1/ABCG16 might compensate for low JA-Ile levels. This idea was triggered by our findings that transcription of *JAT1/ABCG16* was induced in salt-treated roots (Figure 4). A *JAT1/ABCG16:GUS* reporter gene fusion was previously found to be expressed in the vascular system of the roots and, to a lower degree, in leaves [5]. The protein resides in the plasma membrane, where it exports JA out of the cell, but also in the nuclear envelope, where it contributes to the nuclear accumulation of JA-Ile [5]. It was, furthermore, reported that the *jat1* mutant showed reduced expression of *JAZ5* and *JAZ7* in methyl jasmonate-treated leaves and higher susceptibility to the necrotrophic fungus *Botrytis cinereae*. Moreover, root growth was less sensitive to JA-Ile and the JAZ1:GFP fusion protein was not degraded upon treatment of *jat1* roots with 2 µM JA. These phenotypes can be explained by the less efficient intracellular transport of JA-Ile into the nucleus. We therefore speculated that JAT1/ABCG16 might link salt stress with increased COI1-dependent gene expression at low JA-Ile concentrations. However, based on the wild-type-like expression of *JAZ* genes in the *jat1* mutants, this hypothesis was rejected (Figure 5). It is remarkable that JAT1/ABCG16 is required for JAZ1-GFP degradation at 2 µM exogenous JA while being dispensable for NaCl-induced induction of *JAZ* genes at JA levels close to the detection limit. Therefore, it may be speculated that the transporter does not bind to JA-Ile at very low concentrations and that other salt-induced mechanisms sensitize JA-Ile perception and/or signal transduction so that expression of *JAZs* can be triggered.

### 3.4. Possible Activation Mechanisms of the JA-Ile Signaling Cascade at Low JA-Ile Levels

Higher hormone sensitivities of roots as compared to shoots have been observed before. According to textbook knowledge, auxin stimulates growth of roots at 10^6^-fold lower concentrations than stem growth. We also observed lower ABA levels in roots as compared to shoots (Supplementary Figure S2). Plants possess six auxin receptors and 29 co-receptors (Aux/IAA) [31] and 14 ABA receptors and nine co-receptors (PP2C) [32]. Using functional assays in yeast, differences in auxin or ABA sensitivities have been found for different receptor/co-receptor combinations [33,34]. However, these were, at most, in the range of one order of magnitude. The JA signaling pathway is less complex, with only one receptor and 13 co-receptors (13), so alternative mechanisms have to be considered.

One of the possibilities would postulate sensitization of COI1. Extracellular ATP, for example, triggers COI1-dependent JAZ1 degradation even in the *aos* mutant, showing that COI1 can be activated in the absence of the ligand [35]. It is not yet known how the ligand-independent COI1–JAZ interaction is enforced, but second messengers, such as cytosolic calcium, reactive oxygen species and nitric acid, were required. Since these molecules are also generated upon salt treatment [36], it might well be that salt stress primes COI1 for accelerated degradation of JAZ proteins at low JA-Ile levels. Indeed, S-nitrosylation of the auxin receptor TIR1 allows improved (10-fold) interaction with the co-receptor IAA3 [37] and nitrosylation of COI1 might account for the activation of JA signaling by fumigation with NO_2_.

Alternatively, accelerated flux through the JA biosynthesis pathway might be sufficient for increased COI1 activity. In this model, biosynthesis and catabolism of JA-Ile would be enhanced, leading to a higher rate of production of more short-lived JA-Ile, which might stimulate COI1 activity before being catabolized [38]. The first pieces of evidence for such a mechanism were taken from the activation of the JA pathway after fumigation with NO_2_, where increased accumulation of oxidized JA or JA-Ile derivatives was observed during the recovery phase [16].

## 4. Materials and Methods

### 4.1. Plant Material, Growth Conditions and Treatment

All plants used in this study are in the *Arabidopsis thaliana* Col-0 background. Mutant *Arabidopsis* lines were obtained from the Nottingham Arabidopsis Stock Centre *(*NASC), Nottingham University, Nottingham, NG7 2RD, UK: *aos* (SALK_017756), *jar1-1*, *jat1-1* (SALK_119868C) and *jat1-2* (SALK_005006C). The conditional *coi1-16* mutant was provided by John Turner, University of East Anglia, Norwich, UK [39]. Plants were cultivated in a hydroponic growth system at 20 °C with a 12 h:12 h day:night cycle, a photon flux density of 100 µmol photons m^−2^ s^−1^ and 60% relative humidity. Fifteen plants were placed into 15-L black polypropylene containers (Supplementary Figure S1), filled with 12 L hydroponic solution (1.5 mM Ca(NO_3_)_2_, 1.25 mM KNO_3_, 0.5 mM KH_2_PO_4_, 0.75 mM MgSO_4_, 0.1 mM NaSiO_3_, 72 μM Fe(III)-ethylenediaminetetraacetic acid (EDTA), 50 μM KCl, 50 μM H_3_BO_3_, 10 μM MnSO_4_, 2 μM ZnSO_4_, 1.5 μM CuSO_4_ and 75 nM Na_2_MoO_4_). Seeds were sown on pieces of mineral wool (approx. 1 × 1 × 5.5 cm, Grodan) extending into the hydroponic solution and plantlets were covered until the four-leaf stage with small transparent polystyrene cups. Six-week-old plants were treated with 150 mM NaCl for the indicated time spans (addition of 360 mL of a 5 M NaCl stock in water to the hydroponic solution; control: addition of 360 mL water). Roots (emerging from mineral wool) and shoots (whole rosette) were collected separately (Supplementary Figure S1), shock-frozen in liquid nitrogen and stored at −70 °C for further analysis.

For wounding experiments, plants were cut above the mineral wool. Roots separated from the shoots but still connected to the mineral wool holders were squeezed with blunt serrated tip tweezers every 1–2 cm and placed back into containers either with (submersed) or without (air, pseudo-aeroponic) hydroponic solution. After 30 min of incubation, two harvested roots were pooled for one replicate. Non-squeezed but otherwise similarly treated roots served as control material. Separated leaves were squeezed with serrated forceps four to six times across every blade (leaving the midrib intact) and subsequently incubated in petri dishes containing wet filter paper for 30 min in the light. Six leaves from two plants were pooled for one replicate. For the control treatment, non-squeezed leaves from the same plants were incubated under similar conditions as the wounded material.

### 4.2. Quantitative Reverse Transcription (qRT)-PCR

RNA extraction and qRT-PCR analyses were performed as described [40]. Calculations were made according to the 2^−Δ*C*T^ method [41] using the *PP2A* (*At1g13320*) transcript as a reference. Primers serving to amplify and quantify transcript levels are indicated in Supplementary Table S1.

### 4.3. Phytohormone Measurements

Jasmonates were measured by UPLC-ESI–MS/MS using a Waters Acquity I-Class ultra-high-performance liquid chromatography system (Milford, MA, USA) coupled to an AB Sciex 6500+ QTRAP^®^ tandem mass spectrometer (AB Sciex, Framingham, MA, USA), operated in the negative ionization mode. Extraction and chromatographic separation of plant material was carried out as described in [42] using dihydrojasmonic acid (dhJA, 5 ng), JA-Norvaline (JA-Nval, 5 ng) and [18O_2_]OPDA (5 ng) as internal standards. Mass spectrometric analysis was performed under the following electrospray ionization (ESI) source conditions: ion spray voltage was −4000 V, curtain gas was kept at 30 psi and nebulizer gas (GS1) and drying gas (GS2) were adjusted to 50 and 80 psi, respectively, at a temperature of 600 °C. Detection was carried out by selected reaction monitoring (SRM) with a dwell time of 25 ms for each compound and the collision gas (CAD) gas set to 9 psi using the compound-dependent UPLC–MS/MS parameters listed in Supplementary Table S2. Data analysis was performed in Analyst software 1.6 from AB Sciex.

## 5. Conclusions

Transcription of the gene encoding the JA/JA-Ile transport protein JAT1 is highly induced in salt-treated roots. Under these conditions, primary genes of the JA response, namely *JAZ5*, *JAZ7* and *JAZ9*, are induced in a manner that requires JA-Ile and COI1, but JA-Ile levels are close to background. Unexpectedly, JAT1 is not required for *JAZ* expression. The mechanisms that allow the activation of COI1 in the presence of very low JA-Ile levels remain to be explored. In view of low JA or JA-Ile levels found in early land plants, it has to be kept in mind that efficient JA-Ile-dependent responses can happen, even though the ligand is not detected in large amounts [43].

## Supplementary Materials

The following are available online at https://www.mdpi.com/2223-7747/9/12/1635/s1, Figure S1. Hydroponic system; Figure S2. 12OH-JA-Ile and ABA levels in roots and leaves of Arabidopsis thaliana wild-type plants after three hours of NaCl treatment; Figure S3. JAZ transcript levels in salt-treated and wounded roots; Figure S4. 2,3,5-triiodobenzoic acid (TIBA)-induced JAZ10 expression at low JA-Ile levels; Figure S5. Basal JAZ transcript levels in roots of wild-type, aos and coi1 plants; Figure S6: JAT1 transcript levels in root material from wild-type and two different jat1 T-DNA insertion mutants after three hours of either control (−) or NaCl (+) treatment. Table S1. Primers used for qRT-PCR; Table S2. Compound-Dependent LC–MS/MS Parameters, Declustering Potential (DP), Collision Energy (CE) and Cell Exit Potential (CEP).
