# Supplementary material for: Induction of Jasmonoyl-Isoleucine (JA-Ile)-Dependent JASMONATE ZIM-DOMAIN (JAZ) Genes in NaCl-Treated Arabidopsis thaliana Roots Can Occur at Very Low JA-Ile Levels and in the Absence of the JA/JA-Ile Transporter JAT1/AtABCG16
Source: Plants (Basel). 2020 Nov 24;9(12):1635. doi: 10.3390/plants9121635 (PMC7760663; doi:10.3390/plants9121635)
Supplement: Supplementary file 1 [file plants-09-01635-s001.zip › plants-994841-supplementary.docx]

**Induction of Jasmonoyl-Isoleucine (JA-Ile)-Dependent *JASMONATE ZIM-DOMAIN*** ***(JAZ)* Genes in NaCl-treated *Arabidopsis thaliana* Roots Can Occur at Very Low JA-Ile Levels and in the Absence of the JA/JA-Ile Transporter** **JAT1/AtABCG16**

Corinna Thurow^1^, Markus Krischke^2^, Martin J. Mueller^2^, Christiane Gatz^1^

**Supplementary Materials:**

**Figure S1.** Hydroponic system

**Figure S2**. 12OH-JA-Ile and ABA levels in roots and leaves of *Arabidopsis thaliana* wild-type and *aos* mutant plants after three hours of NaCl treatment

**Figure S3.** *JAZ* transcript levels in salt-treated and wounded roots

**Figure S4.** 2,3,5-triiodobenzoic acid (TIBA)-induced *JAZ10* expression at low JA-Ile levels

**Figure S5.** Basal *JAZ* transcript levels in roots of wild-type, *aos* and *coi1* plants

**Figure S6:** *JAT1* transcript levels in root material from wild-type and two different *jat1* T-DNA insertion mutants after three hours of either control (-) or NaCl (+) treatment

**Table S1.** Primers used for qRT-PCR

**Table S2.** Compound-Dependent LC–MS/MS Parameters, Declustering Potential (DP), Collision Energy (CE) and Cell Exit Potential (CEP)

**Figure S1.** Hydroponic system

**(a)**

**(b)**

**Figure S2.** 12OH-JA-Ile and ABA levels in salt-treated roots. (a) Left panel: 12OH-JA-Ile levels in roots and leaves of wild-type and *aos* plants under control conditions and upon three hours of NaCl treatment. Right panel: 12OH-JA-Ile levels in roots and leaves of wild-type plants after two hours of wounding. Data are the mean values +/- SEM from two to five samples. Relative quantification of 12-hydroxy-JA-Ile and 12-carboxy-JA-Ile was performed using deuterated JA-Ile as internal standard. Asterisks denote statistical significance between control and treated samples (unpaired, two-tailed t-test. ***P* < 0.01). n.d., not detected. (b) ABA levels in samples as in (a), left panel. Lowercase letters indicate significant differences (*P* < 0.05) between control and NaCl treatment performed with the same genotype; uppercase letters indicate significant differences (*P* < 0.05) between wild-type and *aos* subjected to the same treatment. Statistical analysis was done for roots and leaves separately (two-way ANOVA followed by Bonferroni’s post-test). n.d., not detected.

Method: Nanoelectrospray (nanoESI) analysis was carried out as recently described. After reversed phase separation of constituents by an ACQUITY UPLC® system (Waters Corp., Milford, MA, USA) equipped with an ACQUITY UPLC® HSS T3 column (100 mm x 1 mm, 1.8 µm; Waters Corp., Milford, MA, USA), phytohormones were ionized in a negative mode and determined in a scheduled multiple reaction monitoring mode with an AB Sciex 4000 QTRAP® tandem mass spectrometer (AB Sciex, Framingham, MA, USA).

Mass transitions and corresponding conditions for determination of ABA and 12OH-JA-Ile

| **MRM Transitions** | | **Analyte** | **DP [declustering potential]** | **EP**  **[entrance potential]** | **CE**  **[collision energy]** |
| --- | --- | --- | --- | --- | --- |
| **Q1** | **Q3** |  |  |  |  |
| 263 | 153 | ABA | -35 | -4 | -14 |
| 269 | 159 | D6-ABA | -30 | -5 | -16 |
| 338 | 130 | 12OH-JA-Ile | -45 | -10 | -30 |

Kusch, S., Thiery, S., Reinstadler, A., Gruner, K., Zienkiewicz, K., Feussner, I. & Panstruga, R. (2019) Arabidopsis *mlo3* mutant plants exhibit spontaneous callose deposition and signs of early leaf senescence, *Plant Mol Biol* 101, 21-40.

**Figure S3.** Quantitative RT-PCR analysis of *JAZ5*, *JAZ7*, and *JAZ10* transcript levels in roots of wild-type and *aos* plants under control conditions (-), upon NaCl treatment for one and three hours (+, left panel) or after wounding (+, right panel) with subsequent incubations for 30 minutes either in hydroponic solution (submerged) or air (pseudo-aeroponic). Data are mean values +/- SEM of two to four samples, each representing pooled root tissue from two individual plants. The *JAZ10* values for the one and three hours control treatment of *aos* plants (marked with crosses) were obtained from one sample, respectively. The other values fell below the detection limit. Statistical analysis was done for the different time points/induction methods separately using two-way ANOVA followed by Bonferroni’s post-test: uppercase letters indicate significant differences (*P* < 0.05) between genotypes subjected to the same treatment; lowercase letters indicate significant differences (*P* < 0.05) between control and treatment performed with the same genotype.

**Figure S4.** 2,3,5-triiodobenzoic acid (TIBA)-induced *JAZ10* expression at low JA-Ile levels. Left panel: Quantitative RT-PCR analysis of *JAZ10* in leaves of wild-type, *dde2-2* (*aos*) and *coi1-t* plants after mock or TIBA treatment. Relative transcript levels were determined using *UBQ5 as a* reference gene. The mean values (+/-SEM) obtained from two to four individually harvested plants are shown. Numbers indicate fold change values after TIBA treatment. Statistical analysis was done using two-way ANOVA followed by Bonferroni’s post-test: uppercase letters indicate significant differences (*P* < 0.05) between genotypes subjected to the same treatment; lowercase letters indicate significant differences (*P* < 0.05) between control and TIBA treatment performed with the same genotype. Right panel: HPLC-MS/MS analysis for the detection of JA-Ile levels. Data are taken from Koster et al., 2012.

Methods: Six-week-old soil-grown plants were either sprayed with 0.1 mM TIBA or 0.1% DMSO or wounded with forceps. Whole rosettes or wounded leaves were harvested for RNA and phytohormone extraction after 8 h of TIBA treatment or 2 h after wounding. FW, Fresh weight; n.d., not detected. For further details see Koster et al., 2012.

Koster, J.; Thurow, C.; Kruse, K.; Meier, A.; Iven, T.; Feussner, I.; Gatz, C. Xenobiotic- and jasmonic acid-inducible signal transduction pathways have become interdependent at the Arabidopsis *CYP81D11* promoter. *Plant Physiol.* **2012,** 159, 391-402.

**Figure S5:** Quantitative RT-PCR analysis of *JAZ5*, *JAZ7*, and *JAZ10* transcripts in root material from wild-type, *aos* and *coi1* mutant plants after three hours of control treatment (same samples as in Fig. 3). Relative transcript levels were determined using *PP2A as a* reference gene. The mean values (+/-SEM) obtained from roots from three to four individually harvested plants are shown. The *JAZ10* value of *aos* plants (marked with a cross) was obtained from one sample, whereas no specific *JAZ10* transcript was detected within the other three analyzed root RNAs. Different letters indicate significant differences (*P* < 0.05) between the genotypes (one-way ANOVA followed by Tukey’s post-test for *JAZ5*, *JAZ7* and *ERF1* expression; unpaired, two-tailed t-test for *JAZ10* expression).

**Figure S6:** Quantitative RT-PCR analysis of *JAT1* transcripts in root material from wild-type and two different *jat1* T-DNA insertion mutants after three hours of either control (-) or NaCl (+) treatment. Relative transcript levels were determined using *PP2A as a* reference gene. The mean values (+/-SEM) obtained from roots from four to five individually harvested plants are shown. Lowercase letters indicate significant differences (*P* < 0.05) between control and NaCl treatment performed with the same genotype; uppercase letters indicate significant differences (*P* < 0.05) between genotypes subjected to the same treatment (two-way ANOVA followed by Bonferroni’s post-test).

**Table S1.** Primers used for qRT-PCR

| **Primer** | **Sequences (5’-3’)** |
| --- | --- |
| JAZ5 | QuantiTect QT00860097 (Qiagen; Hilden; Germany) |
| JAZ7 | QuantiTect QT00716506 (Qiagen; Hilden; Germany) |
| JAZ10 | QuantiTect QT01132999 (Qiagen; Hilden; Germany) |
| JAT1 | QuantiTect QT00792232 (Qiagen; Hilden; Germany) |
| ERF1 | QuantiTect QT00777777 (Qiagen; Hilden; Germany) |
| PP2A qRT For | AAGCAGCGTAATCGGTAGG |
| PP2A qRT Rev | GCACAGCAATCGGGTATAAAG |

**Table S2.** Compound-Dependent LC–MS/MS Parameters, Declustering Potential (DP), Collision Energy (CE) and Cell Exit Potential (CEP)

| **Compound** | **Q1 mass** | **Q3 mass** | **DP (V)** | **CE (V)** | **CXP (V)** |
| --- | --- | --- | --- | --- | --- |
| JA | 209.1 | 59.1 | -35 | -14 | -25 |
| dhJA | 211.1 | 59.1 | -35 | -16 | -15 |
| OPDA | 291.1 | 165.1 | -85 | -26 | -11 |
| [18O2]OPDA | 295.1 | 165.1 | -55 | -26 | -9 |
| JA-Ile | 322.1 | 130.1 | -55 | -38 | -13 |
| JA-Nval | 308.1 | 116.1 | -60 | -26 | -13 |
